# Unsupervised Reference Modeling of Nanopore Signals for DNA/RNA Modification Detection

**DOI:** 10.3390/genes17050525

**Published:** 2026-04-29

**Authors:** Yongji Zou, Mian Umair Ahsan, Kai Wang

**Affiliations:** 1 Center for Cellular and Molecular Therapeutics, Children’s Hospital of Philadelphia, Philadelphia, PA 19104, USA; zaczou@seas.upenn.edu (Y.Z.); ahsanm1@chop.edu (M.U.A.); 2 Bioengineering Graduate Program, University of Pennsylvania, Philadelphia, PA 19104, USA; 3 Department of Pathology and Laboratory Medicine, University of Pennsylvania, Philadelphia, PA 19104, USA

**Keywords:** nanopore sequencing, base modification detection, unsupervised learning, variational autoencoder, anomaly detection

## Abstract

Background: Nanopore sequencing produces ionic current signals that are sensitive to chemical modifications in DNA and RNA molecules. However, accurate modification detection remains challenging due to limited labeled data and variability across experimental conditions. Methods: We present a scalable unsupervised framework for modification discovery that learns reference signal distributions from unmodified sequences using a CNN–Transformer variational autoencoder (VAE). The model is trained on large-scale data via streaming sampling and k-mer-aware soft balancing to ensure robust signal representation. At inference, candidate nucleotides are scored using the VAE reconstruction error, and read-level signals are aggregated to produce site-level modification evidence. Results: On controlled DNA oligonucleotide datasets, models trained on unmodified sequences achieve strong discrimination when evaluated on modified oligos. In contrast, performance decreases in cell line samples when models trained on unmodified whole-genome-amplified (WGA) DNA and in vitro-transcribed (IVT) RNA are evaluated on natively modified (5mC/m6A) data, reflecting the impacts of biological noise and heterogeneity. Despite reduced classification accuracy, site-level anomaly score profiles exhibit peak-like patterns that correspond to known modification-enriched regions. Conclusions: These findings demonstrate the feasibility of large-scale unsupervised reference modeling for de novo modification detection, while underscoring the challenges in translating models built from synthetic oligo datasets into robust genome-wide modification detection.

## 1. Introduction

Chemical modifications of nucleic acids, such as DNA 5-methylcytosine (5mC) and RNA N6-methyladenosine (m6A), are key regulators of genome function and gene expression [1,2]. These epigenetic marks play critical roles in development, cellular identity, and disease, motivating efforts to detect them at scale. Oxford Nanopore sequencing offers a unique platform for modification analysis by measuring ionic current signals as DNA or RNA molecules translocate through a nanopore. Chemical modifications can induce subtle but reproducible perturbations in these signals, therefore enabling the direct and single-molecule detection of diverse modifications without the need for specialized biochemical enrichment [3].

In practice, accurate modification calling remains difficult. Many high-performing approaches rely on supervised learning with curated training labels. However, well-curated modification labels are scarce for many rare chemistries and can be expensive to generate at the scale needed for modern sequencing datasets. Moreover, nanopore signals are affected by the sequence context, read quality, coverage variability, and experimental or computational pipelines, so models trained under one set of conditions can be inadequate when applied to others. These challenges are particularly acute in scientific discovery settings, where the objective is often to prioritize candidate loci for follow-up rather than to produce a definitive genome-wide call set.

### 1.1. Unsupervised Reference Modeling via Reconstruction-Based Anomaly Scoring

In this study, we approach modification discovery as unsupervised reference modeling. Instead of learning a discriminative classifier from modification labels, we learn a model of unmodified nanopore signals and treat deviations as candidates for modification. Concretely, we train a CNN–Transformer variational autoencoder (VAE) on large corpora of unmodified examples, including unmodified synthetic oligos, whole-genome-amplified (WGA) DNA, and in vitro-transcribed (IVT) RNA. The model aims to learn a compact latent representation that supports the accurate reconstruction of canonical signal patterns across diverse sequence contexts. At inference time, we score each nucleotide instance by the reconstruction error, which is the discrepancy between the observed signal and the model’s reconstruction, interpreting larger errors as indicating a higher modification likelihood.

To support practical, genome-scale analyses, we adopt a streaming training strategy: we sample signal windows in a streaming manner from large read collections and optimize the model with stochastic mini-batches. We further incorporate soft k-mer balancing to reduce bias from skewed sequence context frequencies and improve the coverage of rare sequence contexts. Finally, to mitigate noise in single-read signals, we aggregate per-nucleotide anomaly scores into site-level evidence while retaining nucleotide-level resolution for downstream analyses.

### 1.2. Summary of Empirical Findings

We evaluate this approach in both controlled and biological settings. Training on unmodified synthetic sequences and testing on modified oligos yield strong discrimination. In more realistic biological settings, genome-wide performance metrics are weaker, reflecting the difficulty of unsupervised calling under heterogeneous noise sources and variable coverage. Nevertheless, in targeted genomic regions, site-level anomaly score profiles exhibit peak-like patterns that qualitatively track known modification profiles, suggesting that the method can provide useful signals for candidate prioritization and hypothesis generation even when robust genome-wide calling remains challenging.

### 1.3. Contributions

This work offers three contributions.

1. Scalable unsupervised signal modeling. We introduce a streaming CNN–Transformer VAE framework for learning an unmodified reference distribution from large nanopore signal corpora, augmented with on-the-fly sampling and soft k-mer balancing.
2. Reconstruction-based scoring with site aggregation. We propose a practical inference pipeline that uses the reconstruction error as a per-nucleotide anomaly score and aggregates scores into site-level evidence suitable for genome-scale discovery.
3. Evaluation across both controlled modifications and native biological settings. We evaluated our model on controlled modified oligos and on biological DNA/RNA datasets (WGA versus native 5mC; IVT versus native m6A). In addition, targeted regional analyses demonstrated qualitative concordance between anomaly score profiles and known modification-enriched regions.

## 2. Background and Related Work

### 2.1. Nanopore Signal and Modification Calling

Nanopore sequencing measures an ionic current time series as a nucleic acid strand passes through a pore. Chemical modifications perturb this signal in a manner that depends not only on the modified base but also on the local sequence context and upstream processing choices (e.g., normalization, segmentation, alignment of signal to reference). As a result, most computational methods for modified base detection can be viewed as learning (explicitly or implicitly) a mapping from signal + context to either per-read modification probabilities or per-site modification frequencies.

### 2.2. Supervised and Weakly Supervised Callers for Modified Bases

A large body of work focuses on supervised modified base calling, especially for DNA methylation, by training models on datasets with known modification states (often produced via enzymatic treatment, synthetic controls, or matched assays). Early signal-level approaches such as Nanopolish [4] employed hidden Markov models to detect 5mC from aligned nanopore signals. Subsequent deep learning-based frameworks, including DeepMod [5], DeepSignal [6], and related convolutional or recurrent architectures, further improved the sensitivity by directly modeling raw signal features. Systematic benchmarking efforts highlight that accuracy can depend strongly on the genomic context and data characteristics, reinforcing the need for careful evaluation under realistic shifts [7]. More recent implementations such as DeepMod2 [8], ONT’s official basecaller-integrated workflows (Dorado [9]), and related neural network models achieve strong per-read and per-site performance for common marks such as 5mC at CpG sites.

Notably, some supervised approaches do not operate directly on raw ionic current signals but instead use basecalling-derived features such as mismatch rates, insertion/deletion patterns, base quality, or alignment errors as predictors of modification status [10,11]. In these frameworks, chemical modifications are inferred indirectly through systematic perturbations of basecalling behavior rather than explicit signal modeling. Although such approaches may reduce the computational burden and simplify preprocessing, they remain supervised models that rely on labeled training data and may be sensitive to basecaller versions and alignment artifacts.

For RNA modifications, supervised and weakly supervised methods typically rely on either engineered features or model architectures trained with labeled sites (or partially labeled bags of reads). For example, m6Anet [12] uses a multiple-instance learning formulation to call m6A from direct RNA sequencing using a single sample, aiming to infer site-level modification probabilities and stoichiometry without requiring paired controls. Separately, the nanopore official caller Dorado [9] predicts modifications anchored to canonical basecalls or reference alignments and is designed to integrate with ONT pipelines. We have recently benchmarked the performance of these methods using data generated from the latest RNA004 chemistry [13].

### 2.3. “Unsupervised” and Label-Light Approaches: Paired Comparisons and Feature-Based Testing

Alongside supervised callers, several widely used approaches are described as unsupervised or label-light because they do not require the training of a large discriminative model with curated labels.

One family focuses on signal-level testing against expected levels or sample comparison statistics. Tombo [14], for example, provides multiple “modified base detection” strategies, including methods that test for systematic shifts in raw signals and methods that compare samples/conditions after re-squiggling (signal-to-reference alignment).

Another influential family is comparative detection, where modification evidence is derived by contrasting two matched conditions (e.g., WT vs. IVT or enzyme knockout). nanoCompore [15] is a representative framework: it detects modification-sensitive differences by comparing ionic current distributions between a sample of interest and a matched non-modified control, enabling position-level detection without a supervised training set.

These methods are powerful when high-quality paired controls exist and experimental conditions are tightly coupled. However, they can be less convenient when only a single biological sample is available, when controls are expensive or imperfectly matched, or when the objective is to learn a reusable “reference” model from large, heterogeneous unmodified corpora.

### 2.4. Unsupervised Anomaly Detection and Distribution Shift

From a machine learning perspective, modification detection without dense labels is naturally connected to unsupervised representation learning and anomaly detection: learning a model of unmodified signal and score deviations as candidates. In contrast to supervised approaches that depend on labeled data or basecalling error features, reference modeling seeks to learn a reusable generative model of canonical signal distributions.

However, in nanopore settings, this viewpoint faces a distinct challenge: the observed signal distribution can shift substantially across regimes (synthetic oligos vs. whole-genome DNA vs. cellular RNA; WGA vs. native; even different runs/chemistries/basecallers), so “anomalous” may reflect domain shift as much as true chemical modification. Empirically, this motivates both (1) modeling choices that can scale to large unmodified datasets and (2) evaluation protocols that explicitly probe transfer across experimental regimes rather than assuming in-distribution generalization.

Supervised and unsupervised modification-calling strategies offer complementary advantages. Supervised methods generally provide stronger chemistry-specific performance when high-quality labeled training data are available, but they can be sensitive to domain shift across sequencing runs, chemistries, preprocessing pipelines, and biological contexts. In contrast, unsupervised reference modeling does not require dense modification labels and is therefore attractive for discovery-oriented settings or rare chemistries, although its outputs are less directly calibrated and may reflect distribution shift in addition to true modification signals. In practice, supervised methods are often better suited for the definitive calling of well-characterized modifications, whereas unsupervised approaches may be most useful for candidate prioritization, regional discovery, and hypothesis generation when labels are limited or unavailable.

## 3. Problem Setup

### 3.1. Inputs and Notation

We consider nanopore reads aligned to a reference genome (DNA) or transcriptome (RNA). Let *j* index a reference site, i.e., a genomic/transcriptomic coordinate (chromosome/transcript, position, and strand). Let $R_{j}$ denote the set of reads covering site *j*.

For each read $r\in R_{j}$, we construct a fixed-length, per-nucleotide instance centered at the nucleotide aligned to site *j*, represented by a feature tensor

(1) $$x_{r,j}\in R^{L\times C},$$

where *L* is the window length (timesteps/positions in the window) and *C* is the number of feature channels (signal-derived + auxiliary features). The window implicitly encodes the local context, and we additionally associate each instance with an explicit *k*-mer context (derived from the reference) used for sampling/balancing during training.

Our goal is to output

1. An instance-level (per-read, per-site) anomaly score $s_{r,j}$ indicating how much (r,j) deviates from an unmodified reference model.
2. A site-level score $S_{j}$ summarizing modification evidence at reference site *j* by aggregating instance-level scores over reads.

### 3.2. Unsupervised Reference Modeling with a VAE

We assume access to an unmodified training corpus $D_{0}$ (e.g., unmodified oligos, WGA DNA, or IVT RNA), intended to represent canonical (unmodified) signals under a given regime. We train a variational autoencoder (VAE) with decoder $p_{\theta }\left(x|z\right)$ and encoder $q_{\phi }\left(z|x\right)$ to model the distribution of unmodified inputs:

(2) $$\min_{\theta ,\phi }\;E_{x∼D_{0}}\left[L_{\mathrm{VAE}}(x;\theta ,\phi )\right].$$

We optimize the β-VAE objective (weighted negative evidence lower bound, ELBO):

(3) $$L_{\mathrm{VAE}}(x)=\underset{\mathrm{reconstruction}}{\underset{︸}{\ell \;\left(x,\hat{x}\right)}}\;+\;\beta \;\underset{\mathrm{regularization}}{\underset{︸}{\mathrm{KL}\;\left(q_{\phi }\left(z|x\right)\;∥\;p(z)\right)}},$$

where p(z)=N(0,I), $\hat{x}$ is the reconstruction, ℓ(·,·) is the mean squared error (MSE), and β controls the KL term.

### 3.3. Instance-Level Anomaly Scoring

Given a trained model, we compute an anomaly score for each nucleotide instance (r,j) using the reconstruction error:

(4) $$s_{r,j}=\ell \;\left(x_{r,j},{\hat{x}}_{r,j}\right).$$

Intuitively, if $x_{r,j}$ deviates from the learned unmodified manifold (e.g., due to a chemical modification or other systematic perturbation), the reconstruction error tends to increase. This yields a continuous score suitable for candidate ranking.

### 3.4. Site-Level Aggregation and Reliability Summaries

To obtain site-level evidence, we aggregate instance scores across reads covering site *j*:

(5) $$S_{j}=\mathrm{Agg}\left(\left\{s_{r,j}:r\in R_{j}\right\}\right).$$

We consider simple robust aggregators such as the mean and median. In addition to $S_{j}$, we compute coverage and dispersion summaries (e.g., $n_{j}=\left|R_{j}\right|$, standard deviation/MAD of $\left\{s_{r,j}\right\}$, and standard error of the mean) to contextualize confidence as a function of coverage and noise and to enable coverage-aware reporting or thresholding when desired.

### 3.5. Labels for Evaluation

Training is fully label-free. For evaluation, we use reference labels when available, i.e., instance-level $y_{r,j}\in \left\{0,1\right\}$ and site-level $Y_{j}\in \left\{0,1\right\}$ for designed oligos, and high-quality reference tracks (e.g., Dorado calls) as pseudo-labels in biological datasets. We evaluate both instance- and site-level discrimination because they support different use cases: read-level analyses/stoichiometry vs. robust site-level discovery.

## 4. Methods

### 4.1. Overview

**Figure 1:**
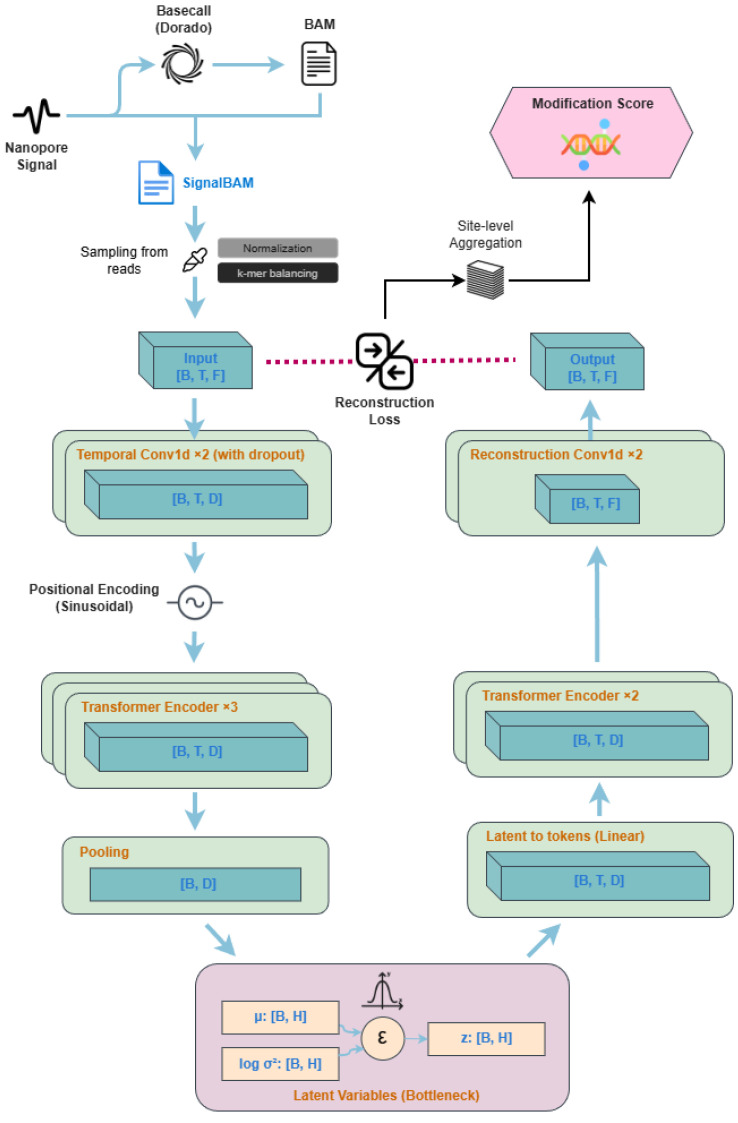
End-to-end pipeline for unsupervised reference modeling and modification scoring from nanopore signals. Raw nanopore current is basecalled (Dorado) to produce read alignments (BAM) and a signal-aligned representation (SignalBAM). Training instances are sampled from reads and converted into input tensors [B,T,F] after normalization and soft k-mer balancing. A CNN–Transformer VAE encodes each window via temporal Conv1D blocks, sinusoidal positional encoding, and stacked Transformer encoders; pools to a latent bottleneck *z* (parameterized by μ and $\log\sigma^{2}$); and decodes back to reconstructed features through latent-to-token projection, Transformer decoding blocks, and reconstruction Conv1D layers to yield [B,T,F]. The reconstruction loss provides a per-nucleotide anomaly score, which is aggregated across reads into a site-level modification score for downstream discovery and candidate prioritization. Notation: B denotes the batch size, T the window length (number of positions in the k-mer context), F the number of input feature channels, D the model (token) dimension, and H the latent dimension.

We learn an unmodified reference distribution of nanopore signal instances using a CNN–Transformer VAE trained on unmodified corpora (Figure 1). Training is performed in a streaming manner by sampling windows dynamically from large aligned read files. At inference time, we use the reconstruction error as an instance-level anomaly score and aggregate across reads to produce site-level evidence.

### 4.2. Input Representation and Feature Construction

For each aligned nucleotide instance (r,j), we extract a fixed-length window centered on the aligned position and construct a feature tensor $x_{r,j}\in R^{L\times C}$.

Signal windowing and normalization: We use a local window around the aligned nucleotide to capture the focal base and neighboring context. The raw current is normalized per read using standard *z*-normalization to reduce run-to-run amplitude variation.

Auxiliary feature channels: In addition to the one-hot base identity, we include event-/window-level summary features, yielding C=11 channels in our primary configuration (base identity, winsorized mean, quartiles, central signal value, RMS energy, and basecaller quality).

Sequence context (k-mers): Each instance is associated with a reference-derived *k*-mer (default k=7) used for soft balancing during streaming. The *k*-mer is not required for VAE reconstruction but helps to reduce sampling bias from highly imbalanced context frequencies.

### 4.3. CNN–Transformer VAE Architecture

We model $x_{r,j}$ using a CNN–Transformer VAE. The CNN encoder captures the local signal morphology, while the Transformer captures dependencies across timesteps within the window.

Encoder: The encoder applies 1D convolutional blocks to map $x_{r,j}$ into a token sequence, adds positional encoding, and passes tokens through a Transformer encoder. A pooling operation produces a fixed-length representation $h_{r,j}$, which parameterizes a diagonal Gaussian posterior:

(6) $$q_{\phi }(z|x)=N\;\left(\mu_{\phi }(x),\mathrm{diag}\left(\sigma_{\phi }^{2}(x)\right)\right).$$

Decoder: The decoder maps *z* back to a reconstructed window ${\hat{x}}_{r,j}\in R^{L\times C}$ via a latent-to-token projection followed by Transformer blocks and a lightweight convolutional head.

Objective: We optimize the β-weighted negative ELBO (Equation (3) in Section 3) with the reconstruction MSE and a β-weighted KL term, using β warm-up during early epochs to improve stability.

### 4.4. Streaming Training with Dynamic Sampling

Nanopore signal corpora are too large to materialize as conventional in-memory datasets. We conduct out-of-core training by sampling training instances directly from aligned read files.

Streaming random sampling: Each optimization step draws a mini-batch by randomly selecting reads and aligned positions and then extracting windows and constructing $x_{r,j}$ online. This produces a stochastic training stream and improves coverage over large corpora.

Soft *k*-mer balancing: To mitigate extreme imbalance in sequence context frequencies, we apply a soft acceptance scheme that downweights overrepresented *k*-mers during streaming. Let $N_{\mathrm{seen}}\left(u\right)$ denote the number of accepted instances so far for *k*-mer *u* (tracked online). We accept a candidate instance with probability

(7) $$p_{\mathrm{accept}}(u)=\min(1,\;\frac{C_{\max}}{N_{\mathrm{seen}}(u)+1}),$$

where $C_{\max}$ is a global soft cap controlling how quickly acceptance decays for frequent contexts. This scheme preserves diversity without imposing hard stratification.

Optimization details: We train using AdamW with standard regularization, β warm-up in early epochs, and early stopping on an unmodified validation subset. The final hyperparameters and model checkpoints are reported in the accompanying code release.

## 5. Experimental Design

### 5.1. Datasets and Evaluation Settings

We perform the evaluation in two complementary settings: (i) controlled synthetic oligonucleotides with designed modification positions and (ii) biological datasets using unmodified proxies for training and native samples for evaluation.

For controlled oligonucleotides (DNA/RNA), we use data released by Oxford Nanopore Technologies [16,17]. We use unmodified oligos as training data and evaluate on modified oligos containing known modification types (e.g., 5mC for DNA and m6A for RNA). Because modified positions are designed, both per-nucleotide and per-site labels are available, enabling the clean benchmarking of sensitivity. The DNA dataset contains 32 short synthetic sequences with an overall modification rate of approximately 20% and explicit site labels for each type of modifications. These engineered patterns differ from the heterogeneous methylation patterns observed in native genomes.

For the DNA oligo datasets, we evaluate de novo modification detection by comparing the reconstruction errors at CpG sites in methylated versus unmethylated oligos. We do not evaluate discrimination between modified and unmodified CpG sites within the same oligo molecule, because the engineered modification sites are separated by only 18 bp. Given the windowed input representation, nearby modifications can influence the local signal context and thus affect the reconstruction error for intervening bases, making within-molecule discrimination less feasible. This design differs from the case of biologically realistic samples, where methylation typically occurs in clustered regions (e.g., CpG islands) but not in regularly spaced engineered patterns. In contrast, evaluation on native HG002 reflects the model’s ability to distinguish modified and unmodified bases within the same biological sample under naturally occurring methylation patterns.

For biological DNA samples, we train on HG002 whole-genome-amplified (WGA) DNA (used as an unmodified proxy) and evaluate on native HG002 DNA containing endogenous methylation (5mC). We report per-site evidence after aggregation to compare with the modification labels generated by the supervised model (Dorado v0.7.2, Oxford Nanopore Technologies, Oxford, UK).

For biological RNA samples, we generate and sequence our own wild-type (WT) and in vitro-transcribed (IVT) data for the HEK293T cell line. We train on IVT RNA (unmodified proxy) and evaluate on WT direct RNA samples containing m6A. We report per-site evidence after aggregation to compare with the modification labels generated by the supervised model (Dorado).

### 5.2. Preprocessing and Instance Construction

All datasets are processed through a common pipeline to obtain aligned read coordinates and per-instance signal windows.

Basecalling and alignment: Reads are basecalled using the best models from Dorado (v0.7.2, Oxford Nanopore Technologies) and aligned to the reference human or synthetic genome/transcriptome using Minimap2. Signal windows are assigned to reference positions using event segmentation and alignment (“re-squiggling”) based on the “move table” output from Dorado.

Signal embedding to BAM: Raw sequencing POD5 files are analyzed and embedded in the aligned BAM files to create an unified data input format to use in model training. Original electric squiggles, as well as general basecalling and alignment information, can be accessed uniformly from the embedded “SignalBAM” file.

Window extraction: For each nucleotide instance from each read, we extract a fixed-length signal window centered on the aligned position and apply normalization and feature construction, as described in the Materials and Methods.

### 5.3. Metrics

We report both instance-level and site-level metrics.

At the instance level (per nucleotide), we calculate the AUROC for discrimination between modified and unmodified instances (where instance labels exist).

At the site level (per site), we calculate the AUROC for site classification after aggregation. We use the average precision (AP/AUPRC) when prevalence is not extreme and labels are reliable.

### 5.4. Regional Case Studies: Concordance with Known Modification-Enriched Regions

To assess whether anomaly scores capture biologically meaningful structures beyond global metrics, we select several genomic regions with known modification enrichment (peak regions from curated Dorado tracks). For each region, we visualize the site-level anomaly score as a genome track and compare its peak-like structure to that of the known modification-enriched signal.

We also report qualitative concordance (visual alignment of peaks) and optionally summarize each region with a simple quantitative statistic, such as (i) the Spearman correlation between smoothed anomaly score and peak tracks across bins or (ii) overlap/enrichment of top-scoring sites within annotated peaks. Within each region, positives are positions inside Dorado peak intervals; negatives are positions outside peaks but within the same region after excluding low-coverage sites.

### 5.5. Implementation Details

We train all models using one A100 GPU with 20 CPUs and roughly 20 epochs on the unmodified training corpus, selecting hyperparameters on a held-out unmodified validation subset.

## 6. Results

We evaluate reconstruction error-based unsupervised reference modeling in (i) controlled synthetic oligonucleotides with designed modifications and (ii) biological datasets where unmodified proxies (WGA DNA; IVT RNA) are used for training and native samples are used for evaluation. Unless otherwise stated, per-site scores are computed by mean aggregation across reads, and per-nucleotide scores correspond to instance-level reconstruction errors, as defined in Section 3.

### 6.1. Controlled DNA Oligos: Strong Per-Nucleotide Discrimination and Near-Perfect Site Aggregation

We train on unmodified synthetic DNA oligos and test on modified oligos with known modified positions. This setting provides clean per-nucleotide and per-site ground truths, enabling the direct assessment of the modification sensitivity independently of biological confounders. In this controlled setting, we compare methylated and unmethylated oligos rather than within-oligo modified/unmodified CpGs due to the close spacing of engineered modification sites (Section 5.1).

**Figure 2:**
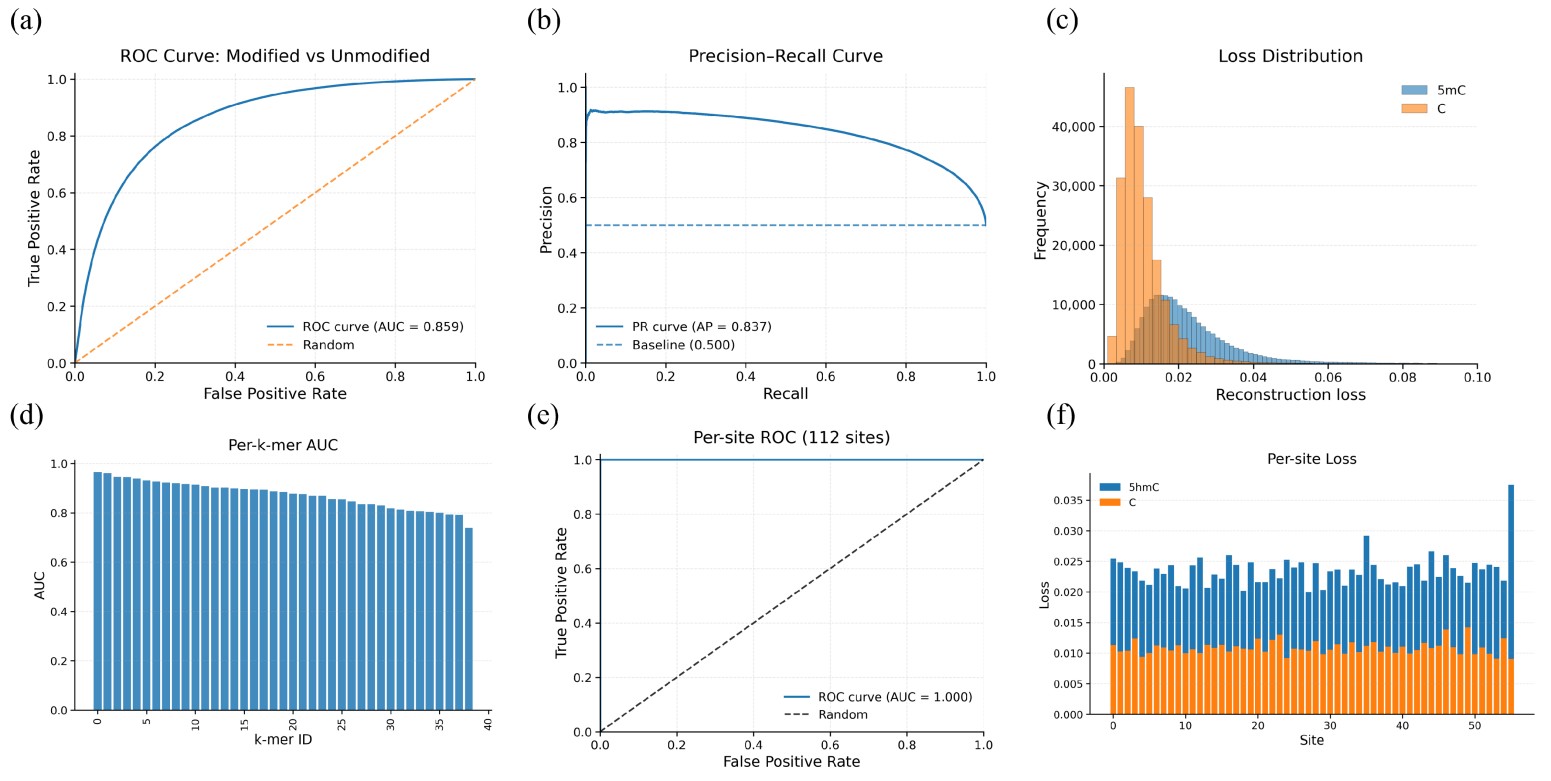
Controlled 5mC oligo evaluation of reconstruction error anomaly scoring at nucleotide and site levels. (**a**) Per-nucleotide ROC curve for modified vs. unmodified instances (AUROC = 0.859; dashed line indicates random baseline). (**b**) Per-nucleotide precision–recall curve (AUPRC/AP = 0.837). (**c**) Distribution of per-nucleotide reconstruction loss for modified (5mC) and unmodified (C) bases, showing a right shift for modified instances. (**d**) Per-k-mer AUROC across sequence contexts, indicating performance variability by local k-mer. (**e**) Per-site ROC computed after aggregating per-nucleotide scores across reads for each site (117 sites; AUROC = 0.991). (**f**) Site-level mean reconstruction loss for modified vs. unmodified sites, illustrating consistent separation after aggregation.

For 5mC (CpG) prediction on oligos, the model achieves strong per-nucleotide discrimination for 5mC at the instance level (AUROC = 0.859; AUPRC = 0.837). Aggregating instance scores to the reference site level yields near-perfect performance (AUROC = 0.991; AUPRC/AP = 0.991), indicating that site-level evidence becomes highly reliable once per-read noise is averaged out (Figure 2).

We further evaluate on additional modified oligos (5hmC) (Supplementary Figure S1). While these chemistries may induce different magnitudes and shapes of perturbations, the same reconstruction error scoring pipeline applies unchanged. The model also achieves strong per-nucleotide discrimination at the instance level (AUROC = 0.854; AUPRC = 0.826). Similarly, aggregating instance scores to the reference site level yields near-perfect performance (AUROC = 0.991; AUPRC/AP = 0.991).

### 6.2. Biological Sample (Human DNA) Analysis: Training on HG002 WGA Sample and Testing on Native HG002 Sample

We train on HG002 whole-genome-amplified (WGA) DNA as an unmodified proxy and evaluate on native HG002 DNA containing endogenous 5mC. Site-level labels for evaluation are derived from Dorado modification calls, which we use as a reference track rather than a definitive ground truth.

**Figure 3:**
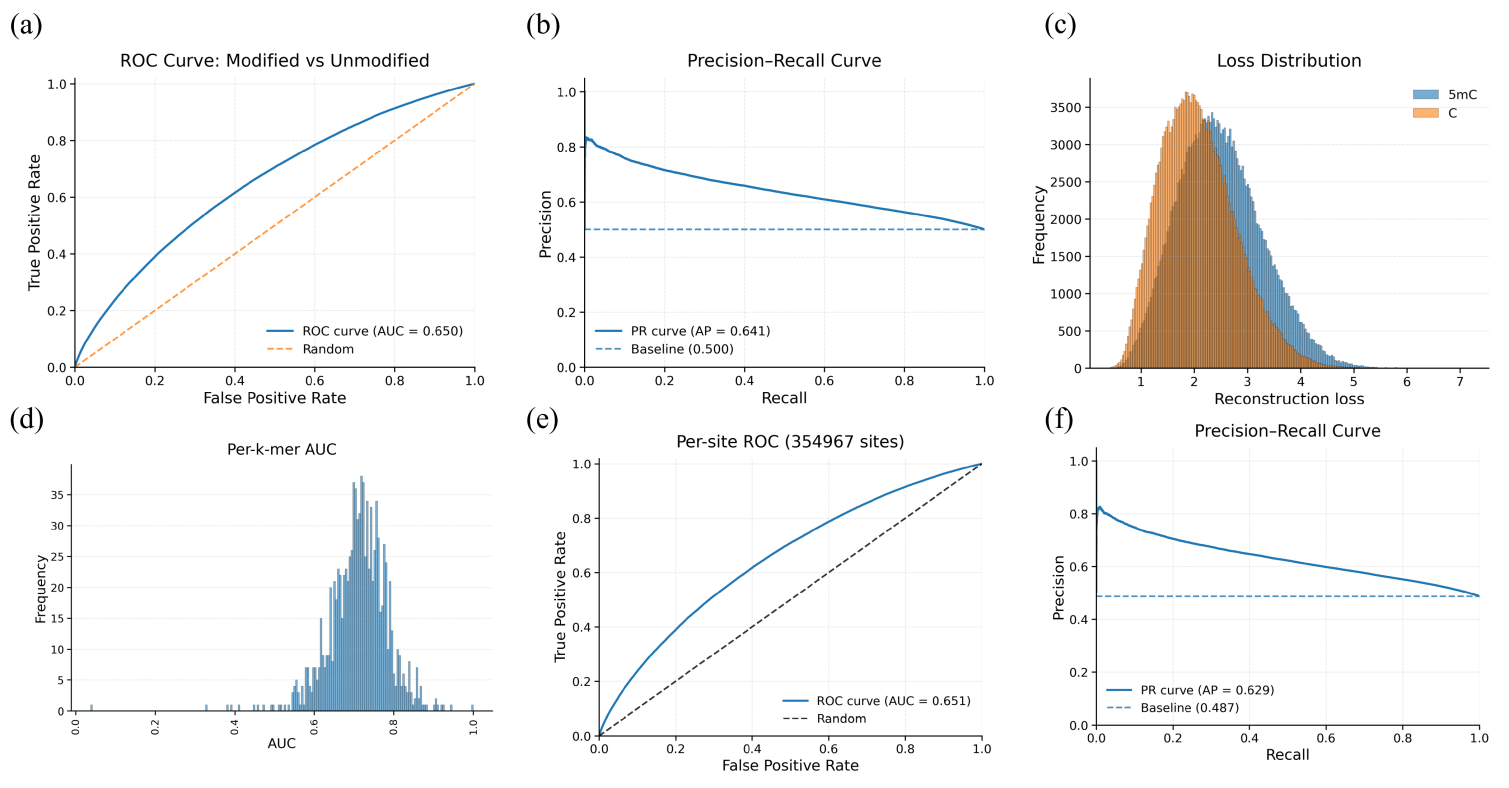
Whole-genome evaluation on HG002: WGA (unmodified proxy) → native 5mC using reconstruction error anomaly scoring. (**a**) Per-nucleotide ROC curve for modified vs. unmodified instances (AUROC = 0.650; dashed line indicates random baseline). (**b**) Per-nucleotide precision–recall curve (AUPRC/AP = 0.641). (**c**) Distribution of per-nucleotide reconstruction loss for 5mC and unmodified C bases, showing partial separation and substantial overlap under biological heterogeneity. (**d**) Histogram of per-k-mer AUROC values across sequence contexts, illustrating context-dependent variability in genome-wide performance. (**e**) Per-site ROC after aggregating per-nucleotide scores across reads for each genomic site (354,967 sites; AUROC = 0.651). (**f**) Per-site precision–recall curve (AUPRC/AP = 0.629), reflecting ranking utility despite modest discrimination at scale.

We evaluate the genome-wide performance of endogenous 5mC prediction on a native HG002 sample. In this more realistic regime, discrimination decreases relative to controlled oligos, showing a performance drop compared to the oligo setting. We find a per-nucleotide AUROC of 0.638 and AUPRC of 0.610. Additionally, we find a per-site AUROC of 0.639 and AUPRC/AP of 0.629 (Figure 3).

The reduction in performance from the controlled (oligo) setting is consistent with additional sources of variability in whole-genome data (such as coverage heterogeneity, broader sequence context diversity, and run/pipeline artifacts). Notably, the model still provides a meaningful ranking of candidate sites, supporting its use in prioritization workflows.

### 6.3. Biological Sample (Human RNA) Analysis: Training on IVT and Testing on Native m6A in HEK293T Cells

We generate direct RNA sequencing data for HEK293T wild-type (WT) RNA and a matched in vitro-transcribed (IVT) RNA dataset. We train on IVT as an unmodified proxy and evaluate on WT, using Dorado m6A calls as the reference track for evaluation.

We first check the genome-wide performance under heavy class imbalance. We observe an AUROC of 0.592 at the per-nucleotide level (Supplementary Figure S2). Because m6A positives are extremely sparse genome-wide, the AUPRC/AP is overdominated by prevalence and label noise.

These results indicate weak signals at the instance level and highlight the difficulty of achieving strong genome-wide calling performance without additional calibration and shift handling in complex RNA samples.

### 6.4. Regional Case Studies: Anomaly Score Tracks Align with Modification-Enriched Peaks

Global genome-wide metrics can understate utility in discovery workflows, where the practical goal is often to prioritize candidate regions and sites rather than to make definitive calls at every position. We therefore examine whether site-level anomaly scores produce biologically meaningful spatial patterns along the genome or transcriptome in regions enriched in modification signals.

**Figure 4:**
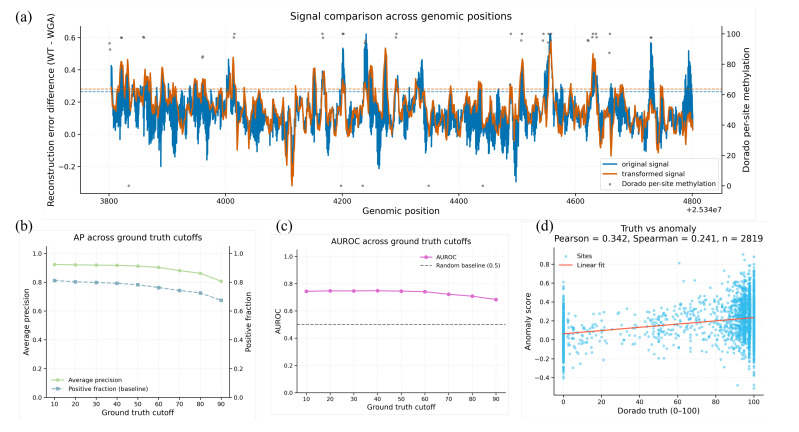
Regional case study showing that site-level anomaly scores track the methylation-enriched structure in native HG002 and remain informative across multiple reference label thresholds. (**a**) Regional signal profile across a representative HG002 locus. The left y-axis shows the reconstruction error difference between native HG002 and WGA (WT − WGA), plotted as the original anomaly signal and a denoised transformed signal; the right y-axis shows Dorado per-site methylation levels (0–100). Horizontal dashed lines indicate representative thresholds used for visualization and region-level evaluation. (**b**) Average precision (AP) for classifying positions within the region across a range of Dorado ground truth cutoffs, with the corresponding positive fraction shown as the baseline. (**c**) AUROC for the same regional classification task across Dorado ground truth cutoffs, demonstrating that discrimination remains above the random baseline over a broad range of thresholds. (**d**) Scatter plot comparing Dorado per-site methylation level and anomaly score across sites in the region (n=2819), showing a modest positive association (Pearson = 0.342; Spearman = 0.241). Together, these analyses indicate that the anomaly score track captures biologically meaningful regional structures and is not dependent on a single arbitrary truth threshold.

For selected regions with strong modification enrichment in the Dorado reference tracks, we visualize the site-level anomaly score profile (mean aggregated across reads) and compare it with the corresponding Dorado per-site methylation signal. In representative regions, the anomaly-score track exhibits a local peak-like structure that broadly follows the reference methylation pattern (Figure 4a), indicating that reconstruction-based reference modeling can recover spatially coherent modification-associated signals even when genome-wide discrimination remains modest.

To quantify this regional concordance more robustly, we evaluate the classification performance across a range of Dorado ground truth cutoffs, rather than relying on a single threshold. The average precision remains consistently above the corresponding prevalence baseline across cutoffs, and the AUROC remains stably above the random baseline over the same range (Figure 4b,c), suggesting that the regional ranking signal is not driven by one arbitrary truth definition. At the continuous level, anomaly scores also show a modest positive association with Dorado per-site methylation values in this representative region (Pearson = 0.342; Spearman = 0.241; *n* = 2819; Figure 4d). Together, these results support the conclusion that the anomaly score track captures biologically meaningful regional structures and may be useful for candidate prioritization and regional discovery analyses, especially for modification types without existing supervised models.

### 6.5. Summary

Controlled oligo experiments demonstrate the strong sensitivity of reconstruction error scoring to modification-induced signal perturbations, with near-perfect performance after site-level aggregation. In biological DNA/RNA settings, genome-wide metrics are weaker under realistic noise and heterogeneity, but regional analyses show peak-like alignment between anomaly score tracks and modification-enriched regions. Together, these results support unsupervised reference modeling as a practical, label-light approach for candidate prioritization and hypothesis generation while motivating future work on calibration and robustness for genome-wide calling.

## 7. Discussion

Our results indicate that reconstruction-based unsupervised reference modeling provides a practical and adaptable framework for nanopore modification discovery. The approach performs strongly under controlled conditions and shows emerging utility in biological datasets when used for prioritization and exploratory analysis rather than definitive site calling.

### 7.1. Current Utility and Use Cases

A key advantage of reference modeling is that it does not require dense modification labels for training. In regimes where labels are unavailable, incomplete, or costly to obtain, reconstruction error scores provide a continuous ranking signal that can guide downstream validation and targeted follow-up. Even when genome-wide discrimination is modest in biological samples, regional analyses suggest that aggregated anomaly score profiles can exhibit spatially coherent patterns that align qualitatively with modification-enriched intervals. This supports discovery-oriented use cases in which a contiguous signal, rather than per-site certainty, is informative—for example, nominating candidate loci, transcripts, or genomic intervals for follow-up experiments, in vitro experiments, or candidate gene/region analyses, where researchers wish to screen for novel or rare modifications without first generating large labeled training datasets.

Although the framework is, in principle, applicable across systems, including animal and human samples, our current biological results indicate that its performance in complex native data remains limited and requires further validation. The approach is potentially extendable to other chemistries when suitable unmodified proxy data exist and the modification induces sufficient signal perturbation—for example, our 5hmC oligo results support this possibility.

### 7.2. Methodological Improvements and Validation Priorities

The performance gap between controlled oligos and biological datasets suggests that covariate shift and heterogeneous noise sources can obscure modification-associated signals. Promising directions include shift-aware normalization (e.g., run/kit/basecaller effects), coverage-aware calibration of score distributions, and aggregation schemes that better accommodate partial occupancy and outlier reads.

For deployment, it is critical that anomaly scores remain stable across sequencing runs, flow cells, and pipeline versions. Multi-run and multi-laboratory evaluations—paired with transparent reporting of preprocessing and parameter choices—would help to quantify reproducibility and establish best practices.

Reconstruction-based scoring is sensitive to the input representation. Changes in basecalling, signal-to-reference mapping, normalization, or feature extraction can shift the learned reference distribution and thus the resulting score scale. We therefore recommend using consistent preprocessing within a study and reporting tool versions and key parameters to support reproducibility.

Elevated anomaly scores can reflect non-biological artifacts (e.g., alignment ambiguity, low-quality reads, signal drift) in addition to chemical modifications. Incorporating QC filters and stratified analyses by read quality, mapping quality, and coverage can reduce spurious discoveries and clarify the conditions under which the method is reliable.

## 8. Limitations

Although controlled oligos exhibit strong sensitivity and site-level aggregation yields near-ceiling performance, genome-wide discrimination in biological DNA/RNA remains comparatively modest. Consequently, the current method is better suited to prioritization and exploratory analyses than to comprehensive genome-wide annotation as a standalone caller.

The current framework consists of chemistry-agnostic anomaly detection rather than chemistry-specific classification. In sequences containing multiple modification types, different perturbations may all elevate anomaly scores relative to the learned unmodified reference. Our current model does not by itself distinguish one chemistry from another. Resolving specific modification identities would likely require additional approaches, such as multi-reference modeling, latent-space analysis, or downstream supervised classifiers, and the detectability is expected to depend on the perturbation magnitude, local sequence context, and domain shift.

Our approach assumes access to an unmodified proxy dataset (e.g., WGA DNA or IVT RNA) and depends on signal-to-reference mapping and normalization decisions. Differences in preprocessing pipelines can alter the learned reference distribution and, in turn, change the anomaly score distributions and thresholds.

Our experiments did not exhaustively assess variability across flow cells, library preparations, basecaller versions, or laboratories. Therefore, claims regarding cross-domain robustness should be interpreted cautiously until broader evaluations are performed.

Finally, performance may vary with the sample composition, ancestry-linked genomic variation, and experimental protocols. Reporting dataset compositions and evaluating across diverse samples can help to identify and mitigate such biases, improving generalizability and responsible use.

## 9. Conclusions

We present a scalable, label-light framework for nanopore modification discovery based on unsupervised reference modeling with a streaming CNN–Transformer VAE. By scoring the reconstruction error as an anomaly signal and aggregating per-nucleotide evidence into site-level summaries, the method achieves strong discrimination in controlled DNA/RNA oligo benchmarks and reveals biologically meaningful, peak-like anomaly patterns in selected genomic regions of wild-type samples. Although genome-wide performance in complex biological datasets remains challenging, these results support reconstruction-based reference modeling as a practical approach for candidate prioritization and hypothesis generation, and they motivate future work on shift handling, calibration, and robust biological validation to enable reliable genome-wide calling.

## Data Availability

Controlled oligonucleotides (DNA/RNA) were downloaded from the Oxford Nanopore Technologies modified base benchmarking release (EPI2ME Blogs) [16,17]. The Oxford Nanopore sequencing data for HG002 are available via the Oxford Nanopore Technologies Open Data Releases [18]. The Oxford Nanopore sequencing data for HG002 with WGA are generated by us and available through the European Nucleotide Archive (ENA) under accession number PRJEB111314. The HEK293T in vitro-transcribed (IVT) RNA and wild-type (WT) direct RNA sequencing datasets were previously generated by us and are available through the European Nucleotide Archive (ENA) under accession number PRJEB80229. The code for implementing the CNN–Transformer VAE framework, data preprocessing pipeline, and analysis scripts is publicly available at https://github.com/WGLab/novamod (accessed on 2 April 2026). Model checkpoints and example configuration files are provided within the repository.

## Supplementary Materials

The following supporting information can be downloaded at https://www.mdpi.com/article/10.3390/genes17050525/s1. Figure S1: Controlled 5hmC DNA oligo validation; Figure S2: RNA biological evaluation.

## References

[1] Delaunay S., Helm M., Frye M. (2024). RNA modifications in physiology and disease: Towards clinical applications. Nat. Rev. Genet..

[2] Jones P.A. (2012). Functions of DNA methylation: Islands, start sites, gene bodies and beyond. Nat. Rev. Genet..

[3] Fu Y., Timp W., Sedlazeck F.J. (2025). Computational analysis of DNA methylation from long-read sequencing. Nat. Rev. Genet..

[4] Simpson J., Workman R., Zuzarte P., David M., Dursi L.J., Timp W. (2017). Detecting DNA cytosine methylation using nanopore sequencing. Nat. Methods.

[5] Liu Q., Fang L., Yu G., Wang D., Xiao C.L., Wang K. (2019). Detection of DNA base modifications by deep recurrent neural network on Oxford Nanopore sequencing data. Nat. Commun..

[6] Ni P., Huang N., Zhang Z., Wang D.P., Liang F., Miao Y., Xiao C.L., Luo F., Wang J. (2019). DeepSignal: Detecting DNA methylation state from Nanopore sequencing reads using deep-learning. Bioinformatics.

[7] Yuen Z.W., Srivastava A., Daniel R., Mcnevin D., Jack C., Eyras E. (2021). Systematic benchmarking of tools for CpG methylation detection from nanopore sequencing. Nat. Commun..

[8] Ahsan M.U., Gouru A., Chan J., Zhou W., Wang K. (2024). A signal processing and deep learning framework for methylation detection using Oxford Nanopore sequencing. Nat. Commun..

[9] Oxford Nanopore Technologies Dorado. https://github.com/nanoporetech/dorado/.

[10] Liu H., Begik O., Lucas M.C., Ramirez J.M., Mason C.E., Wiener D., Schwartz S., Mattick J.S., Smith M.A., Novoa E.M. (2019). Accurate detection of m6A RNA modifications in native RNA sequences. Nat. Commun..

[11] Jenjaroenpun P., Wongsurawat T., Wadley T.D., Wassenaar T.M., Liu J., Dai Q., Wanchai V., Akel N.S., Jamshidi-Parsian A., Franco A.T. (2021). Decoding the epitranscriptional landscape from native RNA sequences. Nucleic Acids Res..

[12] Hendra C., Pratanwanich P.N., Wan Y.K., Goh W.S.S., Thiery A., Göke J. (2022). Detection of m6A from direct RNA sequencing using a multiple instance learning framework. Nat. Methods.

[13] Zou Y., Ahsan M.U., Chan J., Meng W., Gao S.J., Huang Y., Wang K. (2025). A comparative evaluation of computational models for RNA modification detection using nanopore sequencing with RNA004 chemistry. Brief. Bioinform..

[14] Stoiber M., Quick J., Egan R., Eun Lee J., Celniker S., Neely R.K., Loman N., Pennacchio L.A., Brown J. (2017). De novo Identification of DNA Modifications Enabled by Genome-Guided Nanopore Signal Processing. bioRxiv.

[15] Leger A., Amaral P.P., Pandolfini L., Capitanchik C., Capraro F., Miano V., Migliori V., Toolan-Kerr P., Sideri T., Enright A.J. (2021). RNA modifications detection by comparative Nanopore direct RNA sequencing. Nat. Commun..

[16] Stoiber M. (2024). Modified Base Best Practices and Benchmarking. EPI2ME Blog (Oxford Nanopore Technologies).

[17] Stoiber M. (2025). RNA Modified Base Best Practices and Benchmarking. EPI2ME Blog (Oxford Nanopore Technologies).

[18] Talenti A. (2023). Sequencing Genome in a Bottle samples. EPI2ME Blog (Oxford Nanopore Technologies).

