# Supplementary material for: Unsupervised Reference Modeling of Nanopore Signals for DNA/RNA Modification Detection
Source: Genes (Basel). 2026 Apr 29;17(5):525. doi: 10.3390/genes17050525 (PMC13205325; doi:10.3390/genes17050525)
Supplement: Supplementary file 1 [file genes-17-00525-s001.zip › genes-4204679-supplementary.pdf]

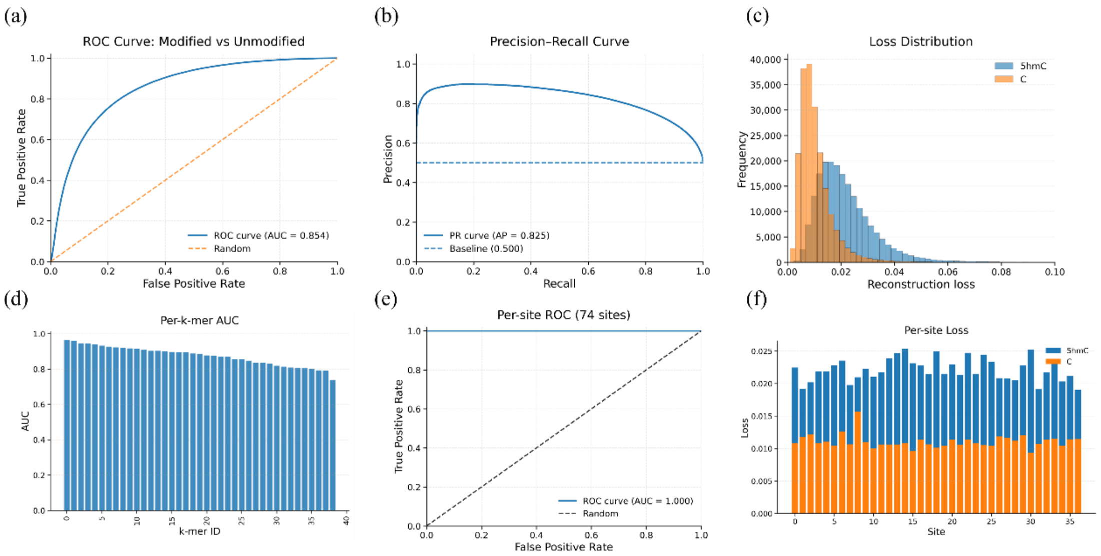

Figure S1: Controlled 5hmC DNA oligo validation. A CNN–Transformer VAE is trained on unmodified DNA oligos and evaluated on a matched set of unmodified versus 5-hydroxymethylcytosine (5hmC)–modified oligos using reconstruction error as the anomaly score. **(a)** Per-nucleotide ROC curve for modified vs. unmodified instances (AUROC = 0.854; dashed line indicates random baseline). **(b)** Per-nucleotide precision–recall curve (AUPRC/AP = 0.826). **(c)** Distribution of per-nucleotide reconstruction loss for 5hmC and unmodified C bases, showing a right-shift for modified instances. **(d)** Per-k-mer AUROC across sequence contexts, indicating context-dependent performance. **(e)** Per-site ROC after aggregating per-nucleotide scores across reads (74 sites; AUROC = 1.000). **(f)** Site-level mean reconstruction loss for modified vs. unmodified sites, illustrating consistent separation after aggregation.

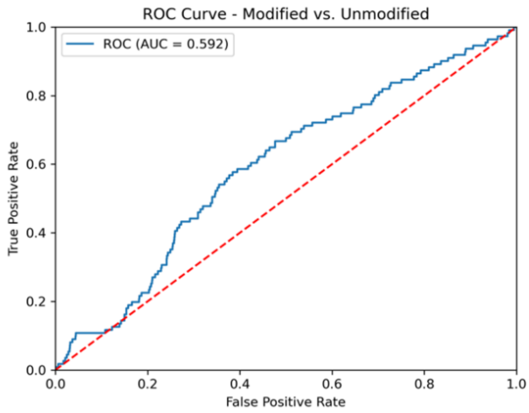

Figure S2: RNA biological evaluation. A CNN–Transformer VAE is trained on in vitro transcribed (IVT) RNA as an unmodified proxy and evaluated on wild-type (WT) RNA, with modified/unmodified labels derived from Dorado's per-site m6A calls. Per-nucleotide ROC curve for distinguishing Dorado-labeled modified vs. unmodified instances based on reconstruction-error anomaly scores (AUROC = 0.592; dashed line indicates random baseline).
